# Letter to the Editor regarding “Diagnosis, treatment, and outcome prediction of non-convulsive status epilepticus in unconscious patients in intensive care units” by Willems et al.

**DOI:** 10.1186/s42466-026-00512-5

**Published:** 2026-07-14

**Authors:** Moisés León-Ruiz, Carlos Castañeda-Cabrero, Julián Benito-León

**Affiliations:** 1 Department of Neurology, Severo Ochoa University Hospital, Avda. de Orellana s/n, Leganés, Madrid, 28914 Spain; 2 Faculty of Medicine, Alfonso X El Sabio University, Leganés, Madrid, Spain; 3 Department of Clinical Neurophysiology, Puerta de Hierro University Hospital, Majadahonda, Madrid, Spain; 4 Department of Neurology, 12 de Octubre University Hospital, Madrid, Spain; 5 Research Institute (i+12), 12 de Octubre University Hospital, Madrid, Spain; 6 Department of Medicine, Faculty of Medicine, Complutense University of Madrid, Madrid, Spain; 7 Centro de Investigación Biomédica en Red Sobre Enfermedades Neurodegenerativas (CIBERNED), Madrid, Spain

## Dear Editor,

We read with great interest the narrative state-of-the-art review by Willems et al. about diagnosis, treatment, and outcome prediction of non-convulsive status epilepticus (NCSE) in unconscious patients in intensive care units (ICUs) [1].

The authors address the reliable initial diagnosis and further monitoring of NCSE using the Salzburg criteria and the 2HELP2B score, therapy options beyond current guideline recommendations, and prognosis assessment using established scores and metrics, such as STESS, SACE, EMSE, and END-IT. With succinct tables and illustrations, comprehensive insights are shown to provide clear guidance for daily clinical practice [1].

They conclude that NCSE is a common and complex disease entity observed in the ICU that requires dedicated and specialised diagnostics, therapy, monitoring, and outcome assessment. Nevertheless, the availability of continuous (cEEG), quantitative electroencephalography (qEEG) in the ICU and expertise in its interpretation are limiting factors in many clinical settings, especially because of a reduced or complete lack or reimbursement for c/qEEG [1].

However, we would like to add some concerns about the pediatric NCSE:

First, seizures are included among the most common pediatric emergencies, and both seizures and SE, frequently warrant critical care resources in pediatric emergency departments (PEDs) [2]. NCSE is more difficult to recognize than convulsive SE because it usually expresses solely as an altered mental status with little or no visible motor activity [3], delaying diagnosis and treatment, and raising the risk of unfavourable outcomes [3].

Second, it is recommended to perform an EEG after the first unprovoked seizure (US), including the onset as SE, to define the recurrence risk and the diagnosis of epileptic syndrome, start antiseizure medication (ASM), and avoid misdiagnosis of epilepsy, especially in prolonged psychogenic non-epileptic seizures (PNES). Of note, children (8–14 years) with PNES can be misdiagnosed as SE in up to 3% of boys and 7% of girls [4]. If EEG is performed within the first 16 h after seizure onset, when there is the highest probability to show epileptiform EEG patterns compared to 20.2% if performed after (*p* < 0.001) [4].

Third, SE is a frequent pediatric neurological emergency that needs rapid management. NCSE is common in pediatric inpatient settings (including the ICU). Incidence can be up to 46% of critically ill children, and in those with suspected NCSE, it was established in 14% by EEG [5].

Finally, a recent study using a point-of-care EEG (pocEEG), prospectively included patients with isolated altered mental status (AMS), active seizures, and other neurologic events. It used a customized, two-channel pocEEG system for bedside neuromonitoring in a tertiary PED. The system comprised a mobile monitor equipped with an EEG module and four frontotemporal electrodes with one channel per hemisphere. Of 5 children diagnosed with NCSE, 4 were identified in the PED by using pocEEG. In one case, standard EEG 23 h after presentation revealed focal seizures; retrospective review of the initial pocEEG showed an ictal-interictal continuum. Finally, although all cases followed convulsive seizures, pocEEG assisted in detecting NCSE in children with unexplained AMS and no seizure history, allowing earlier diagnosis and treatment of pediatric NCSE when standard EEG is unavailable [2].

To conclude, the work by Willems et al. [1] represents an important step toward highlighting the importance of the availability of cEEG, qEEG in the ICU and to know that expertise in its interpretation are limiting factors in many clinical settings [1]. We would like to add that early recognition and intervention of NCSE (a time-critical diagnosis with potential adverse outcomes) in the adult and pediatric populations, as well as close follow-up and monitoring, are critical for mitigating morbidity and mortality. Moreover, pocEEG may constitute a useful diagnostic adjunct for timely seizure detection and clinical decision-making. Further larger studies to better understand the exact pathophysiology, epidemiology, and risk factors, and to develop new and effective therapeutic interventions in both populations, are warranted.

## Data Availability

No datasets were generated or analysed during the current study.
